# Science and Politics

**Published:** 1877-09

**Authors:** 


### Editorial.

### SCIENCE AND POLITICS.

In our issue for October of last year, we published a somewhat extended editorial notice of the Chicago Botanical Garden, in which, speaking for the medical profession of the city, we expressed our satisfaction with the work already accomplished by the Directors, and commended the object they had in view to all who were interested in the study of science.

Since that date, those who have visited the south-eastern corner of the northern of the two south parks, have noticed with pleasure the labor expended in the cultivation of the more than one thousand beds—fifteen acres—of plants arranged in their natural order, as well as the luxuriant development of the flora in the four hot-houses.

And here we have to present the resolutions lately passed by the Board of South Park Commissioners, which are subjoined:

WHEREAS, The great stagnation in all industrial enterprises of our country has made taxation at this time specially burdensome, therefore the Commissioners called for a smaller sum of money for park purposes than the Board had done in former years, rendering a corresponding curtailment in our expenditures absolutely necessary; therefore,

*Resolved*, That the further operations of the Botanical Department are hereby suspended, and the garden placed under the general management of the Floral Department of the park, with direction to the Superintendent to take charge of and preserve all its valuable plants, flowers, seeds, and other property, until a change in the times shall warrant this Board in expending the necessary means to carry out such an enterprise.

*Resolved*, That the Secretary is hereby instructed to send a copy of this preamble and resolution to the President and Directors of the Botanical Gardens, and to express the thanks of this Committee to the same, and particularly to Prof. H. H. Babcock for the great energy and skill manifested by him, as well as the regrets of this Board at being obliged to suspend their good work.

To this summary action of the Park Commissioners the gentlemen entrusted with the management of the Botanical Gardens have filed the following protest:

CHICAGO, August 6.—The undersigned, who were invited and appointed by the Board of South Park Commissioners to undertake the establishment, management, and direction of a Botanical Garden in the South Park, do hereby earnestly protest against the action of the said Board as set forth in the preamble and resolutions copied above, for the following reasons; viz.,

*First*. Because said action was taken at a meeting from which we, as well as the public and reporters for the press, were excluded.

*Second*. Because we have reason to believe that said action was not, as stated in the preamble, based upon considerations of economy in the expenditure of public money, but is the result of machinations whose object has been the defeat of the enterprise, against which we have been obliged to contend from the inception of the Garden, and which the Board has taken no efficient means to prevent.

*Third*. Because said action, though nominally a suspension, is virtually the abandonment of the Chicago Botanical Garden as a scientific establishment.

*Fourth*. Because such action of the Board in appropriating to general park purposes material solicited and contributed for scientific uses, obtainable from no other sources, and by no other means than those adopted, is a gross breach of faith and honor, rendering us powerless to fulfill the promise, made with the approval of the Board, to make returns in kind for the contributions we have received from all parts of the civilized world, and for which it is impossible for the Board to render an equivalent.

*Fifth*. Because we regard said action of the Board of South Park Commissioners as a serious blow to the advancement of science in Chicago and the Northwest—a bar to the inauguration of any similar enterprise in our section, and a blot upon the fair fame of our city and country; so that hereafter no resident of Chicago can visit the galleries of other countries without a blush of shame at the ignoble termination of our own, after the expenditure of so much money, and when in the full tide of successful progress.

H. N. Hibbard, William Bross,
H. H. Babcock, E. H. Sargent,
A. E. Ebert.

No part of the protest filed by the Directors of the Botanical Garden deserves greater consideration than that which describes the evident breach of faith and honor, which the discontinuance of the Garden necessitates. The existence and remarkable development of the latter, has been rendered possible only by an adoption of the most generous system of exchanges on the part of numerous foreign institutions as well as individuals who, in many cases, were ready to wait for a return till the Chicago enterprise had become fully established. The truth is that the Chicago Garden has found some of its warmest advocates and friends outside of its own home. And what is the deplorable result? A deliberate repudiation of obligation, a repudiation, precisely such as those to which the foreign capitalist is disposed to make a pointed reference, whenever he is solicited to invest in American securities!

Let us look such contingencies as these squarely in the face. The little lesson we have to learn and impress upon the scientific world is this, that in a Republic such as ours, art, literature and science can never flourish under the protection of political influence. However brilliant may have been their conquests under the patronage of such men as the Princes of Florence, the Louis of France, and even the Pontiffs of the conservative Church of Rome, we must admit that in this country the upas-tree of "politics" casts a deadly shadow upon that which aspires to bloom beneath its branches. What species of fruit has ripened to maturity beneath its shadow in the past? In the field of literature we can point to the various government reports, which are generally sold for waste paper; and the two latest productions of the pen of the American politician, "Why we laugh" and "How to play poker." In the art studies of the government, we have to be satisfied with such grotesque absurdities as can be seen in the rotunda of the National Capitol. Look at the bold design impressed upon the coins of the country; analyze the architectural effects in the average government building, and in what respect has science been benefited by its occasional sustentation by the state? The Agricultural Bureau, according to the *New York Nation*, of August, has been "for years, the laughing stock of the newspapers" of the land. Fortunately for the name of the medical profession in this country, the general suspicion of political influence has never menaced the Surgeon Generals' Office at Washington, and the results have been correspondingly superb. The latest production of politics in medicine is the recently published Government Report on the *Cholera Epidemic of* 1873, and of this work it is scarcely necessary to remark that when Dr. Max von Pettenkofer finished with it in the London *Practitioner*, (Feb. and March, 1877), the only part of it left was the tail—Dr. Billings' excellent Bibliography.

We have no objection to urge against the constitution and functions of our own newly-organized State Board of Health, being unwilling to obstruct its first steps by adverse criticism, but content ourselves for the present, with remarking that in general, an alliance between medicine and the state has resulted, at least in this country, disastrously to the former.

We cannot, therefore, but deplore the issue in the matter of the Chicago Botanical Garden, regarding it in the language of the protest, as a "blot upon the fair fame of our city and country." Let us, however, learn the lesson well. With us, science and politics must be eternally divorced. For the children of such a union will be suckled, like Romulus and Remus, by a she wolf—a wolf that resembles the *lupus exedens* of the pathologist, in that it is ready to destroy all the beauty and excellence to which they may aspire.

---

The Illinois State Board of Health is now issuing licenses to the physicians of this State, according to the new law, which was published in our last number. Many physicians misunderstand the scope and intent of this law, which we wish might have been a better one. The various western States are agitated over special legislation on the subject of our profession. Doctors are greatly divided in their opinions as to the feasibility of any special legislation for themselves. Some contend that no law is needed to regulate the practice of medicine and surgery. Others as stoutly clamor for protection against charlatanism. It will not be five years before every western State will have a medical bill. California has the best of those we know, and it seems to work well. Illinois has one now, and we must make the best of it. Many of our Illinois physicians seem not to understand it.

They feel astonished that *physicians with diplomas* should be passed upon by a Board created by law; they feel rebellious, pugnacious. Some Doctors insinuate that the Colleges are the gainers by this law. Some urge one objection and others urge another, while but few seem to comprehend the wonderful advantages resulting from this law, imperfect as it is, which are these:

A. This new law gives all educated physicians the power to protect themselves and elevate the standard of the profession, which they never before had. In less than twelve months every County Clerk in Illinois will have recorded the license of all physicians practicing in his county. Every new comer can be investigated, by any man choosing to ascertain if the former is registered, by inspecting the County Clerk's record. If he be registered, well and good; if not, the County Attorney, in obedience to instructions from the State Board, will prosecute the new comer, who must show that he is a graduate, or "move on." Thus the profession can keep out of Illinois all uneducated, ungraduated men.

B. After January 1st., prox., physicians, in collecting fees in courts, must show that THEY are law-abiding citizens, in that THEY have qualified by conforming to the laws of the State—in short, that they are licensed practitioners of medicine and surgery. Failing in this, they will suffer immensely in their suit and be greatly embarrassed.

C. All advertising specialists and traveling quacks can be suppressed, by licensed physicians entering complaint to the

State Board against them, alleging that they are violating the "code," and the Board will at once recall licenses issued to those thus giving offense. As long as the earth stands there will be charlatans, and no law can be framed that will completely annihilate them. Quacks are such by NATURE. Doctors must always expect to be pestered by this sort of vermin. This new law places in our hands, *for the first time in our State history*, the possibility of dealing summarily with all forms of quackery. The "codes" of all schools are agreed as to what is "professional conduct."

From the foregoing, it can be seen that if the profession throughout Illinois will cheerfully come forward and array themselves on the side of law and order, give their hearty and united moral support to this Board of Health, the standing of physicians will be greatly enhanced thereby, and they only will be the gainers. On the general principle of opposition to special legislation, the Colleges, so far as is known, were opposed to the bill, and the fact that eight separate medical bills were introduced in the legislature last winter, and but *one* (and an imperfect one) passed, shows that there was no endeavor on the part of Colleges to secure the enactment of this law.

The best method for Illinois physicians to pursue, is to comply with the law, by sending to the President of the Board, Dr. J. H. Rauch, No. 202 State Street, Chicago, for a blank affidavit, and executing it at once. If the physician has *not* practiced in Illinois ten years, he *must send his diploma with the executed affidavit* to some member of the board, accompanying it with the license fee of one dollar ($1.00), and he will, in a few days, receive his diploma and certificate in due form and order. While it is not necessary for practitioners of over ten years standing to send their diplomas with their affidavits, still it is better to send their parchment in, so that they can be *recorded* as physicians with diplomas. It places them in a better light before the public and gives them a higher standing.

Any physician having practiced in Illinois more than ten years, will save trouble by sending his certificate. The ten year clause in the law *does not absolve any physician from*

*making affidavit.* The Board, fortified by the opinion of the Attorney General, will, after January 1st, 1878, *compel* every old practitioner who claims exemption by the ten year clause, to make affidavit that he has practiced ten years. The Board does not *know* that A, B, or C, has been in practice ten years or longer; it *knows* only what is corroborated by affidavits. Thus it will be seen every practitioner has to comply with the law. The Board now *asks* every doctor to comply with the law. After January first proximo, it will *demand* what is now *asked*, and, not receiving it, will inflict the penalties. Now is the time for the profession in this State to rise in their might and unite against quackery in all of its forms. It can successfully be done by nothing short of a united front, and combined effort and determination.

